# Are We Overlooking Harms of BDDE-Cross-Linked Dermal Fillers? A Scoping Review

**DOI:** 10.1007/s00266-024-04262-0

**Published:** 2024-08-06

**Authors:** Marta Wojtkiewicz, Albert Stachura, Bartłomiej Roszkowski, Natalia Winiarska, Karolina Kazimierska, Kamilla Stachura

**Affiliations:** 1 Department of Methodology, Medical University of Warsaw, 1B Banacha Street, 02-091 Warsaw, Poland; 2 National Medical Institute of the Ministry of the Interior and Administration, 137 Wołoska Street, 02-507 Warsaw, Poland; 3 Dr Stachura Clinic, Jagiellońska 87 Street, 70-437 Szczecin, Poland

**Keywords:** BDDE, 1,4-Butanediol diglycidyl ether, Dermal fillers, Hyaluronic acid

## Abstract

1,4-Butanediol ether (BDDE) is widely used as a cross-linker for hyaluronic acid in dermal fillers. The purpose of this scoping review was to determine the state of knowledge about the behaviour of cross-linked substances and safety of BDDE application. The rationale behind the review came from the clinical experience of one of the authors (KS), who noticed adverse reactions after BDDE-linked hyaluronan application. The scoping review was conducted according to PRISMA-ScR guidelines. Out of 399 articles, 52 met the inclusion criteria. Data on study design, sample/population, aims, methodology, outcomes and funding were extracted. Results were charted according to 6 subtopics: rheological properties, hydrogel stability, BDDE toxicity, immunogenicity, tissue interactions and clinical studies. In vitro, cross-linked hydrogels were characterized as effective fillers in terms of viscosity and elasticity; however, previously uncharacterized by-products of the cross-linking reaction were found. Most in vivo studies reported increased dermis regeneration, vascularization and anti-inflammatory cytokine release after implantation of BDDE-cross-linked substances. In clinical studies, BDDE was shown to sensitize subjects to 1,6-hexanediol ether and other substances found in epoxy resin systems. Occupational dermatitis and hypersensitivity reactions were documented. Our review shows that BDDE may have long-term adverse effects, which are overlooked in the safety assessment of fillers. Reviews on BDDE conducted so far have mostly been sponsored by the industry, potentially leading to incomplete reporting of adverse effects. A review of the occurrence of allergic reactions after commercial dermal filler use and analysis of possibly harmful by-products of BDDE hyaluronan degradation are needed.

*Level of Evidence III* This journal requires that authors assign a level of evidence to each article. For a full description of these Evidence-Based Medicine ratings, please refer to the Table of Contents or the online Instructions to Authors www.springer.com/00266.

**Supplementary Information:**

The online version contains supplementary material available at 10.1007/s00266-024-04262-0.

## Introduction

1,4-Butanediol diglycidyl ether (BDDE) is a compound renowned for its cross-linking properties, with uses ranging from biomaterials to dermal fillers based on hyaluronic acid (HA) [1–6]. BDDE-cross-linked fillers were first introduced in Europe in 1996 and in the USA in 2003 and have become increasingly popular since [2]. According to the most recent survey conducted by the International Society of Aesthetic Plastic Surgeons, HA injections were the second most performed non-surgical treatment worldwide in 2022, with over four million procedures and a 15% rise since 2018 [7]. BDDE is the predominant cross-linker used for HA, with all but three dermal fillers registered in Europe and all those in the USA containing BDDE [2]. Since BDDE usage for other medical procedures remains marginal, a comprehensive understanding of the dynamics and interactions of BDDE within tissues is crucial, especially for dermal fillers.

While BDDE is widely used, its long-term safety is not well documented. Although the metabolism of BDDE in vitro is thoroughly studied, the in vivo behaviour of cross-linked gels, especially in humans, remains only partially understood. Allergic reactions associated with BDDE have been reported [8–11], pointing to interactions with the immune system. Concerns about carcinogenicity and long-term toxicity have also been raised [12, 13]. Only two reviews relating to BDDE as a cross-linker have been published, one dating back to 2013 and funded by Allergan, and the other sponsored by Teoxane, a producer of BDDE-cross-linked fillers, raising concerns about the quality of the evidence appraisal [2, 14].

In this scoping review, we aimed to examine the extent, range and nature of literature relating to BDDE and its cross-linking capacity with a focus on its potential toxicity, tissue interactions and harms. Summarizing the existing evidence may help determine the value of undertaking a systematic review of long-term harms associated with the use of BDDE-cross-linked fillers. Moreover, we aimed to identify research gaps in existing literature and suggest future directions in the field. In our work, we also wanted to outline contradicting evidence, as well as highlight potential conflicts of interests of authors researching the topic of BDDE.

## Materials and Methods

The protocol was created in accordance with the PRISMA-ScR guidelines [15]. Included were studies reporting in vitro*, *in vivo*,* or clinical investigation of the physicochemical properties, toxicity, immunogenicity and tissue interactions of BDDE alone or as a HA cross-linker. We excluded reviews, letters to editors and studies in which BDDE was mentioned solely as a cross-linker, without data on its characteristics. We also excluded studies not concerning soft tissue engineering and those focusing on collagen cross-linking, as we aimed to focus on BDDE in the context of dermal fillers based on hyaluronic acid—its most prevalent use.

All data were collected from publicly available sources. Two bibliographic databases: PubMed and Scopus, were screened on 18.03.2023 using the keywords: BDDE, 1,4-Butanediol diglycidyl ether. The combination for record extraction was [(“BDDE” or “1,4-Butanediol diglycidyl ether” not “Boron-doped diamond electrode” not “Body dysmorphic disorder examination”)]. Only studies in English were retrieved, and no time restrictions were set. The reference lists of identified studies were searched for additional articles. Duplicate studies were removed using Rayyan [16].

The studies were first screened based on the title and abstract by two reviewers and then selected for full-text review. Decisions were recorded in Rayyan, and disputes were resolved by discussion to reach a consensus or with a third researcher. The full text of each study was then assessed by two reviewers, and disagreements regarding inclusion were discussed as described above. One meeting of the whole team was organized to review included studies and establish the charting categories. Eventually all studies were sorted into six key themes: HA cross-linking and rheological properties, HA stability and degradation, BDDE toxicity, BDDE immunogenicity, BDDE–tissue interactions and clinical data.

We extracted the following data from the full texts of each article: study design (in vitro*/*in vivo/mixed methods/clinical), population (gel characteristics for in vitro studies, cell/animal type and number for in vivo studies, number of participants in clinical studies), methodology, key findings and funding source. Data were collected by four researchers who worked in pairs, independently extracting data from each article. Comprehensive results of data charting can be found in appendix (Supplementary Tables 1–6).

## Results

**Fig. 1:**
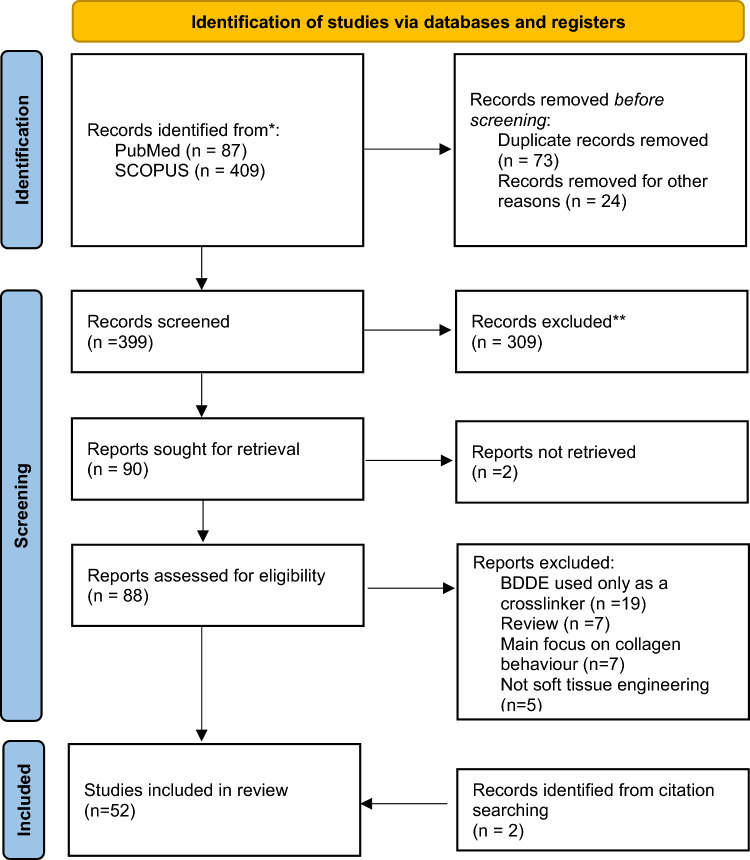
PRISMA 2020 flow diagram for the scoping review process

**Fig. 2:**
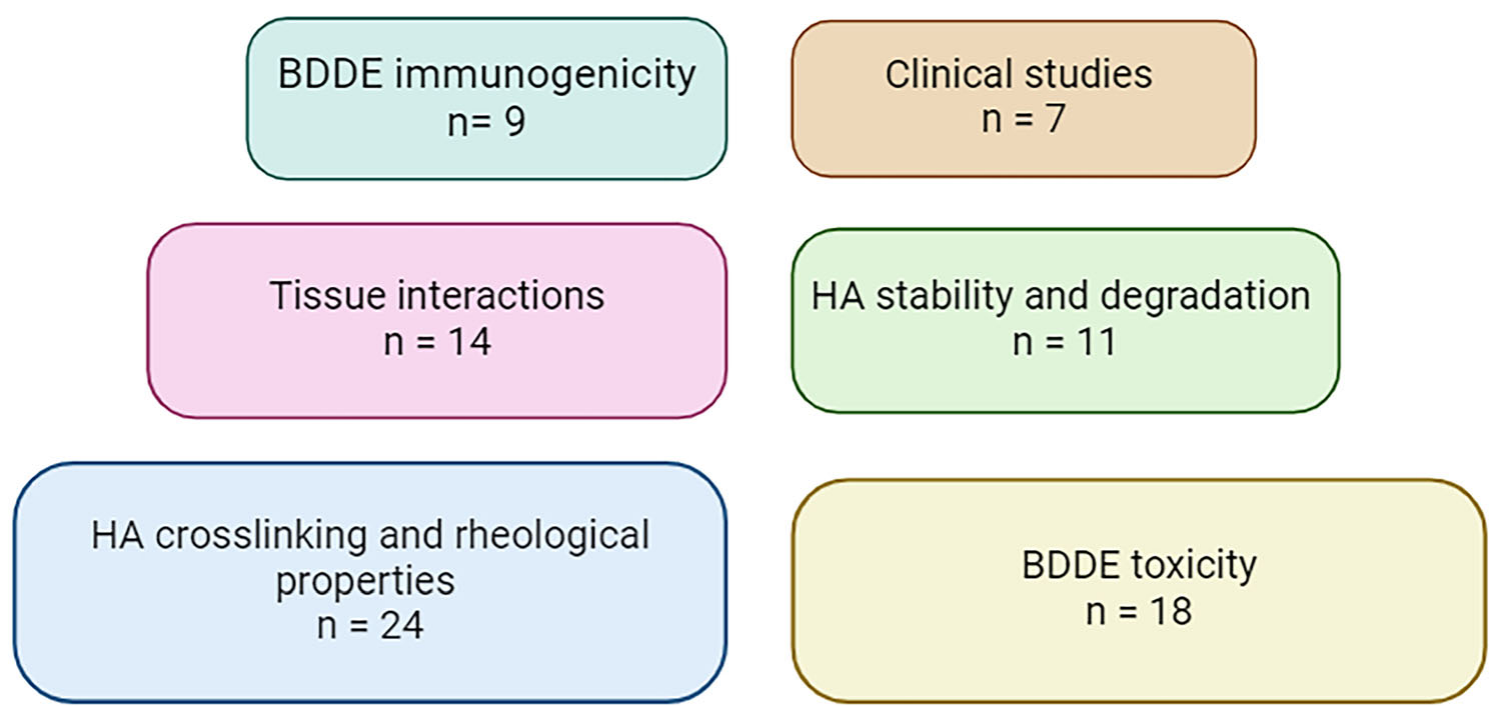
Number of studies within each subtopic. Several studies were classified under more than one subtopic

Of 399 studies, 52 were eligible for inclusion (Fig. 1), of which 27 had in vitro design, 10-in vivo*,* 8 used mixed methods and 7 were clinical studies. All articles were charted based on the main topics covered (Fig. 2). Descriptive results are outlined in Supplementary Table 1.

### HA Cross-linking and Rheological Properties

Key rheological characteristics are viscosity and elasticity. The viscosity (loss modulus—G’’) represents the capacity of a substance to flow freely from a needle and elasticity (storage modulus—G’) signifies the ability to resist deformation when acted upon by a force. The latter is also the best predictor of in vivo hydrogel residence [17]. For HA hydrogels cross-linked with BDDE, G’ is generally higher than G’’ [18].

Cross-linking with BDDE makes gels more rigid and allows them to retain their form when acted upon by gravity. Higher BDDE concentrations are positively associated with the level of HA modification and inversely associated with the swelling degree. Swelling is also proportionately dependent on HA molecular weight [19]. High levels of cross-linking may decrease swelling capacity, as the number of coiled HA chain interactions rises [20–26]. When HA concentration in a hydrogel increases, HA becomes exposed to a larger number of BDDE molecules leading to subsequent formation of covalent bonds. Swelling degree decreases with rising HA concentration peaking at 10% and then gradually increasing [27]. The matrix structure of HA hydrogels cross-linked with BDDE resembles a spider’s web, which expands more slowly when the cross-linking degree is high (a few days) compared with non-cross-linked HA, which expands more and faster (within 2 days) [18, 28]. For cross-linked hydrogels, water absorption rate is higher at the initial stages and declines over time [29]. Compared with gels cross-linked with PDE or PEG, those cross-linked with BDDE exhibit lower levels of intrinsic viscosity and gels cross-linked with PEGDE have increased elasticity compared with BDDE when the same molarity is used [30].

BDDE is highly pH-sensitive, and the cross-linking process is temperature-dependent [31, 32]. Hydrogels produced at lower temperatures are less degradable, have more efficient and denser cross-linking and constitute a more elastic final product, though with reduced water absorption potential [32]. Unfortunately, some studies do not provide data on factors like temperature or cross-linking time, which impact the cross-linking efficiency. Additionally, the concentration of BDDE is most often determined prior to the cross-linking, and the degree of modification is not reported after the reaction has taken place.

### HA Stability and Degradation

Stability of the HA hydrogels increases nonlinearly with an increasing cross-linking degree, achieved with higher BDDE concentration, and decreases over time [20, 23, 28]. At supra-optimal BDDE concentrations (> 5%), the degradation time may be extended. A denser hydrogel structure prevents hyaluronidase from infiltrating and slows the degradation process [32]. Similarly, adding amino acids to HA chains improves hydrogel stability [33].

Manufacturing influences hydrogel stability. Lower temperatures improve enzymatic resistance (likely due to lower swelling capacity) and degradation rate is dependent on the methods used for mixing the gel [32]. From the HA concentration range of 7–14%, optimal stability is achieved at 10% [27]. Compared with PEG and PDA, BDDE shows a better enzyme-mediated degradation resistance [34]. Storage methods impact hydrogel stability. For up to 30 days, a stable degradation rate over a two-month period after implantation is maintained. After 45 days, the degradation rate increases almost twofold, and elasticity decreases [17].

Interestingly, multiple small doses of hyaluronidase seem to be more effective in dissolving hydrogel after 120 min than a single high-dose administration. Short injection intervals (5 min) maximize the optimal enzymatic degradability, likely because of a loosening of the three-dimensional hydrogel structure [35].

### BDDE Toxicity

Selected reports provided no evidence for BDDE cytotoxicity in fibroblasts [19, 32, 36, 37], smooth muscle cells, vascular endothelial cells and Schwann cells up to 72 h in vitro [38]. The cross-linker increases cellular proliferation in fibroblasts and adipose-derived mesenchymal stem cells [39, 40]. In corneal epithelial cells, however, 0.1% BDDE was highly cytotoxic, killing > 20% of cells within 48 h [41]. One study assessed the effect of BDDE and PEGDE on keratinocytes. The use of BDDE was associated with lower cell viability, higher LDH release, higher cellular reactive oxygen species production, higher expression of tumour necrosis factor (TNF)-*α* and interleukin (IL)-1*β*, as well as higher mitochondrial membrane disruption and COX-2 protein expression. Most effects were dose-dependent [13]. On the contrary, another study showed BDDE induced a lower cytotoxicity index than PEGDE or PPDGE when tested on human epithelial MiaPaCa-2 cells [42].

Two large in vivo studies in a murine (*n*=500) and *D. melanogaster* (*n*=1841) model investigated mutagenicity and systemic effects of BDDE [12, 43]. Oral administration of 2.84% BDDE to flies resulted in a lethal mutation rate of over 1%—a finding associated with a significant number of chromosomal translocations [43]. In mice, cutaneous exposure to 0.05% and 0.2% BDDE over 103 weeks did not adversely affect survival. The total number of tumours and irritant lesions was slightly higher when using BDDE than in the control group, though differences were not statistically significant [12].

In a clinical study, where BDDE-HA was applied to humans after lumbar discectomy and compared to sodium carboxymethylcellulose, a statistically significant higher incidence of adverse effects related to the nervous system was noted, mainly pareaesthesia and hypoaesthesia [44].

Two studies reported previously undescribed by-products of the BDDE-HA cross-linking reaction [17, 45]. 1,4-Butanediol di(propan-2,3-diolyl) ether (BDPE) was shown to be cytotoxic only as a residue, not when chemically bound to HA [17]. Hydrogels produced in alkaline conditions and at a high temperature promote the formation of a "propene glycol-like" compound, the toxicity of which is currently unknown [45]. To fully understand their properties, further investigation of these substances is required.

### Immunogenicity

The immunogenicity of BDDE has been studied in vivo and in clinical studies investigating its role as an allergen. In vivo, BDDE cross-linked HA shows significant anti-inflammatory activity decreasing TNF-*α*, IL-6 and IL-8 cellular secretion [20]. Macrophages exposed to HA-BDDE hydrogels show variable NO production, with lowest expression at moderate BDDE concentrations compared with either higher or lower concentrations [25]. Cross-linking with BDDE decreases the immune response to implants, resulting in lower lymphocyte proliferation and antibody levels [46]. Interestingly, in one study the longest cross-linking duration was correlated with the lowest immune response, suggesting an immunomodulating role of BDDE [47]. No in vivo study reported any significant inflammatory reaction, and the degree of immune response did not depend on BDDE concentration, though data on this are limited. This lack of adverse effects has been observed both after subcutaneous and peri-neural application [17, 24, 38, 48]. However, no in vivo study examined the immunogenicity of sole BDDE.

In clinical studies BDDE has been suggested as a potential source of late-onset immune reactions, possibly due to the degradation of cross-linked substances over time and the release of toxic by-products [8–11]. Adverse reactions were also observed in several clinical studies examining epoxy resin systems, in which BDDE often acts as a diluent. BDDE caused allergic reactions when applied in patch tests, as well as exhibiting the ability to permeate rubber gloves and induce contact dermatitis [9]. Furthermore, cross-reactions were observed between BDDE and other molecules with similar chemical structure, such as 1.6-hexanediol ether, which shows that there is potential for sensitization to BDDE due to contact with a wide range of substances [11]. BDDE was also identified as the main cause of occupational allergic contact dermatitis in some cases. Geier’s study showed that BDDE reactions were more common than reactions to a similar substance (PGE), suggesting a higher sensitization potential [8].

### Tissue Interactions

In vivo, BDDE-cross-linked implants influence extra-cellular matrix remodelling and increase neovascularization, collagen fibre deposition and dermis regeneration. This effect is greater in samples linked with BDDE compared to both other cross-linkers and un-cross-linked controls [32, 49–51]. However, one study reported conflicting results after the application of a BDDE-linked hyaluronan membrane to rabbit cornea, with decreased vascularization and fibrosis and increased reepithelization [41].

In several studies, BDDE-cross-linked hyaluronan has a positive effect on wound healing, which is in line with in vitro studies, where cross-linked products increase cell growth and adhesion [37]. These properties are influenced by the HA concentration and the cross-linking temperature [32]. Metalloproteinase expression rises after BDDE-linked implant application, suggesting an increased tissue reorganization and integration with the ECM [50]. In two studies, a fibrous capsule formation was noted around the implant, which may impact BDDE–tissue interactions, slowing implant degradation but also possibly impairing its incorporation into the surrounding tissues [32, 47].

### Clinical Studies

Only two clinical reports were directly related to BDDE composition in HA fillers [1, 3]. One study examined Uma Jeunesse, which featured novel BDDE cross-linking technology, and Juvéderm, a product from Allergan’s line, by injecting them into facial folds. Juvéderm lost more than 50% of its initial aesthetical effect by the 6th month, while Uma Jeunesse experienced less than a 20% reduction in its benefits at the 9-month follow-up [1]. Another study compared TEOSYAL RHA, a product with lower BDDE concentration, to Juvéderm Volift and Ultra. The findings revealed that RHA fillers had similar effectiveness but lasted longer, providing more satisfaction over an extended period [3].

## Discussion

We showed that the majority of the BDDE literature relates to its cross-linking capacity, HA hydrogel rheology and potential toxicity studied in vitro or in vivo. Interestingly, in vivo investigations revealed no significant pro-inflammatory activity of BDDE, and some pointed to its potential beneficial immunomodulating function. In contrast, a series of observational studies showed BDDE may cause late-onset immune reactions, likely associated with its degradation or release of previously undescribed by-products. It seems BDDE may also cross-react with other antigens. Most included studies were not funded by the industry.

The rationale behind this review came from the clinical experience of one of the authors (KS), who has been working with dermal fillers for 15 years and has been training physicians in volumetric techniques for 10 years. In several patients, she noticed massive swelling and tissue thickening at the site of injection (particularly tear troughs, peri-ocular areas, nasolabial folds, cheek bones, chin) occurring a few months following the use of BDDE-cross-linked HA dermal filler. These events were usually associated with upper respiratory tract infections or allergic dermal reactions to different antigens. The symptoms were transient and reacted to anti-histaminic treatment. Additionally, in some cases rapid neovascularization and formation of telangiectasias were noted. No such observations were made in patients treated with HA fillers cross-linked differently. Reviewed evidence suggests BDDE may be linked to both cross-reactivity and enhanced local production of growth factors, possibly responsible for the creation of new blood vessels.

A dermal filler cross-linked with BDDE (Restylane^®^) was first approved by FDA in 2003 based on a report of a randomized controlled trial done on 138 patients [52]. It was concluded that “benefits of the use of the device (…) outweigh the risk of illness or injury (…)” even though patients could only report pre-defined self-perceived adverse effects up to 14 days post-treatment and safety case report forms were filled by an unmasked physician. No hypersentivity reactions were observed, despite 2 cases of contact dermatitis (though it is unclear in which study arm). Given that dermal filling is neither a life-saving nor a significantly health-improving intervention, it seems reporting of harms should be stricter to avoid potential bias and ascertain reliable results.

A highly cited Allergan-funded review from 2013 suggested 15 years of data provide assurance for safety of BDDE use in dermal fillers [14]. It did not, however, review the methodology used in clinical studies, in which BDDE-cross-linked fillers were used. It may be speculated that most such reports focused on aesthetic outcomes and could have been underpowered to detect harms (especially if occurring rarely). Another issue may be that harms in such reports are overlooked or underreported, as many studies are funded by the industry. To understand potential rare harms associated with BDDE-linked fillers, a systematic review of randomized clinical trials should be conducted [53]. Alternatively, a well-designed case–control study could help identify associations between BDDE use and rare harms. However, it must be noted that studies comparing the safety of BDDE to PEG, a novel cross-linker, offer only preliminary results regarding the superiority of PEG. The first filler cross-linked with PEG was introduced in 2014, almost two decades later than those using BDDE, and data regarding its safety in vivo and long-term viability in humans are therefore scarce [54].

Our study has limitations. Firstly, its focus was to gain a broad understanding of the BDDE literature. By searching superficially and with general keywords, we aimed to identify different study designs to see the breadth rather than depth of the evidence base underlying current assumptions behind BDDE safety statements. Therefore, evidence on harms reported in clinical trials using commercial products cross-linked with BDDE was not identified in the search. However, to appraise evidence on such harms a systematic review with subsequent meta-analysis would be required. Our study showed that such a review may indeed be needed. Furthermore, out of 52 studies included in the review, more than half (27) are investigations carried out only in vitro, whereas only 15 studies had an in vivo component. Data regarding rheological properties, cross-linking conditions and gel stability are extensive and consistent across studies, while data concerning the cytotoxic potential and immunogenicity of BDDE in vivo are not so abundant and results are at times conflicting. Finally, out of the seven clinical studies identified, only two regard hyaluronan cross-linked with BDDE in dermal fillers. As such, our review shows a disproportion between study design prevalence and potential for more research regarding the live tissue response to BDDE.

Secondly, a scoping review may be subjective and prone to missing significant findings, but we followed a structured process to identify eligible studies and worked iteratively as a team to solve any disagreements, as well as chart the data in an informative way.

## Conclusion

The main aim of this scoping review was to assess the state of existing research regarding the safety of BDDE in dermal filler use. In vitro, BDDE-cross-linked gels had optimal rheological features for soft tissue filling, which were impacted by cross-linking conditions. The cytotoxic effect of BDDE differed among studies, but several investigations noted its immunomodulating role and a positive effect on wound healing. Clinically, BDDE was characterized as an allergen, causing contact dermatitis and cross-reactions with other substances used industrially. In the review, we showed that knowledge of the long-term effects of BDDE and its behaviour in vivo is lacking, suggesting that both late-onset harms and previously unknown by-products of the cross-linking reaction between hyaluronic acid and BDDE need further analysis.

## Supplementary Information

Below is the link to the electronic supplementary material.

Supplementary file1 (DOCX 193 kb)

## Data Availability

Data used for this study can be accessed in the full-text articles included in the review. Additional data can be obtained from the corresponding author upon reasonable request.
