# Correction: Are We Overlooking Harms of BDDE-Cross-Linked Dermal Fillers? A Scoping Review

**DOI:** 10.1007/s00266-024-04337-y

**Published:** 2024-08-21

**Authors:** Marta Wojtkiewicz, Albert Stachura, Bartłomiej Roszkowski, Natalia Winiarska, Karolina Kazimierska, Kamilla Stachura

**Affiliations:** 1 Department of Methodology, Medical University of Warsaw, 1B Banacha Street, 02-091 Warsaw, Poland; 2 National Medical Institute of the Ministry of the Interior and Administration, 137 Wołoska Street, 02-507 Warsaw, Poland; 3 Dr Stachura Clinic, Jagiellońska 87 Street, 70-437 Szczecin, Poland

**Correction to: Aesthetic Plastic Surgery** 10.1007/s00266-024-04262-0

The original online version of this article was revised to note in the Abstract and Discussion sections that the rationale for the scoping review came from the clinical experience of coauthor Kamilla Stachura.

The original article has been corrected.

