# Supplementary material for: Are We Overlooking Harms of BDDE-Cross-Linked Dermal Fillers? A Scoping Review
Source: Aesthetic Plast Surg. 2024 Aug 6;48(23):5147–54. doi: 10.1007/s00266-024-04262-0 (PMC11739315; doi:10.1007/s00266-024-04262-0)
Supplement: Supplementary file 1 — Supplementary file1 (DOCX 193 kb) [file 266_2024_4262_MOESM1_ESM.docx]

## **Supplementary table 1. Hyaluronic acid (HA) crosslinking & rheological properties.**

| **Author & year** | **Population/ sample** | **Design** | **Study aim** | **Methodology** | **Key findings** | **Funding** | **Reference** |
| --- | --- | --- | --- | --- | --- | --- | --- |
| Al-Sibani, M., 2017 | HA hydrogels with different initial concentrations of HA | *In vitro* | Assessing the impact of different initial concentrations of HA (ranging from 7.0% to 14.0%) on the cross-linking efficiency with 2.0 % BDDE solution and the stability of the hydrogel. | The effect of HA initial concentration on cross-linking efficiency was evaluated through various analytical tests, including in vitro degradation rate, swelling behavior, scanning electron microscope (SEM) and Fourier-transformed infrared (FT-IR). | Incorporation of a larger HA content in the reaction medium resulted in more exposure of HA chains to BDDE molecules, thus more covalent ether bonds were formed. The swelling ability of tested hydrogels steadily decreased with the increase of the initial concentration of HA until 10.0 %, and then began rising again, following a U-curve trend. | Vivatis Pharma (Hamburg, Germany) – supplied hyaluronic acid | ^1^ |
| Baek, J., 2018 | HA hydrogels crosslinked with BDDE at different temperatures | *In vitro* | Determing the impact of crosslinking HA with BDDE at different temperatures on hydrogel behaviour. | Properties of obtained HA hydrogels were evaluated in terms of the rheological behavior and swelling ratio. The cross-linking temperature ranged from 3°C to 37°C | By cross-linking at lower temperatures, the cross-linking efficiency can be enhanced and the cross-linking density can be improved. HA hydrogel becomes more elastic when formed at lower temperatures, however, the water absorption is gradually reduced with falling temperatures. | Public (China) | ^2^ |
| Chang, L., 2019 | HA hydrogels cross-linked with BDDE | *In vitro* | Explore the effects of different BDDE concentrations on HA hydrogel properties. | BDDE was added at concentrations 5, 10, 15, 20, 25, 30, and 35μL/mL. The studied outcomes were swelling degrees and the modification degree assessed with mass spectrometry, and morphology assessed with scanning electron microscopy. | The swelling degree is inversely associated with BDDE concentration, with an especially marked difference between 5 and 10 μL/mL, then following a more stable, linear trend. T-MOD, c-MOD and p-MOD increase with increasing BDDE concentrations, which is associated with a denser hydrogel network structure and more robust backbone. | Public (China) | ^3^ |
| Choi S.C., 2015 | BDDE crosslinked HA hydrogels | *In vitro* | Assessing the impact of different amounts of BDDE on the mechanical and biocompatibility properties of a HA hydrogel. | 26.5 mg/mL HA polymers were dissolved in 0.1 M NaOH solution at room temperature. 0.5 to 10 ppm BDDE was added as a crosslinker. | The percentage of crosslinking increased by 1.3% when the concentration of BDDE was increased from 0.5 to 10ppm. The elasticity of the gels increased correspondingly. | Public (Republic of Korea) | ^4^ |
| Cui, Y.J., 2012 | HA cross-linked hydrogels (CHA) | *In vitro* | Differentiating the physicochemical properties of various crosslinked hydrogels. | HA hydrogels cross-linked with BDDE, polyethylene glycol (PEG) poly diglycidyl ether (PDE) or adipic acid dihydrazide (ADH) were compared | The intrinsic viscosity of resultant gels is as follows: PDE-CHA > PEG-CHA > BDDE-CHA > ADH-CHA. | Henan University of Technology (China) | ^5^ |
| Dias, J.R., 2017 | Crosslinking gelatin mesh with varied BDDE concentrations. Human dermal neonatal fibroblasts (hDNF). | *In vitro* | Investigation of physicochemical and biological properties of BDDGE-crosslinked electrospun gelatin meshes. | Gelatin was crosslinked in situ with BDDGE at different concentrations (2, 4 and 6 wt-%) and incubation time-points (24, 48 and 72h) at 37ºC. | 4% and 6% BDDGE (both incubated for 72h) provided gelatin fibers with a high crosslinking degree and pore diameters of 339±91 and 276±88 nm.  A concentration 4% BDDGE resulted in the best combination of mechanical properties. | Public (Portugal) | ^6^ |
| Kaya, G., 2021 | HA hydrogels with different concentrations of BDDE (1-5%) | *In vitro* | Investigation of the rheological, swelling and injectability properties of HA hydrogels | BDDE at different concentrations (1, 2, 3, and 5% in respect to the final volume) was added to the HA solution and stirred for an additional 5 min. The cross-link ing reaction was then performed at 45 ⁰C for 4 hours. | The rheological characteristics of hydrogels were enhanced with increasing crosslinker concentration: the elastic modulus of the hydrogels ranged from 280 Pa to 990 Pa, while the complex viscosities were between 42 Pa.s and 190 Pa.s at an oscillation frequency of 1 Hz. This leads to the conclusion that modifying the concentrations of BDDE enables a precise tuning of rheological properties. | Public (Turkey) | ^7^ |
| Keizers, PHJ., 2018 | Dermal fillers - Hyacorp, Rofil, Hyamax products | *In vitro* | Establishing a methodology to analyze the crosslinking grade of HA based filler products on a routine basis. | An analytical method was developed and validated to identify HA-BDDE-based fillers and to quantify their modification and crosslinking grade | The composition of Hyacorp products deviated from the producer’s declaration and the products were found to contain elevated modification and crosslinking grades. This likely contributes to the heightened risk of adverse effects. | No funding to report | ^8^ |
| Kim, DJ., 2021 | Hyaluronic acid membrane (HAM) crosslinked with BDDE | *In vitro* | Developing a HA membrane crosslinked with BDDE for treating ocular surface diseases and injuries | The water absorption and swelling ratio, chemical composition, optical transmittance and residual BDDE of membranes were determined. Karl-Fischer titration was used for determining water content, spectroscopy (IR, UV, gas), SEM and chromatography | BDDE-linked membranes displayed a high capacity for water absorption, and good optical transmittance. The pore size in the membrane decreased after crosslinking. <0.5ppm residual BDDE was found in the HAM after washing, thus below toxic levels as per regulations. | Public (Korea) | ^9^ |
| Kumar, V., 2013 | Acellular components from swim bladder of fish (Labeo rohita) | *In vitro* | Comparing the physical properties of crosslinked samples. | The swim bladders were crosslinked with 0.6% glutaraldehyde (GA), 1% BDDGE, and 1% 1-ethyl-3 -(3-dimethylaminopropyl) carbodiimide (EDC).The physical properties were determined by SDS-PAGE analysis. | The moisture contents of GA, BDDGE, and EDC treated acellular swim bladder were significantly (P<0.05) lower as compared to control. | Public (India) | ^10^ |
| La Gatta, A., 2020 | HA hydrogels | *In vitro* | Modifying the molecular weight of hyaluronic acid and assessing its impact on the properties of HA-BDDE crosslinked hydrogels. | Cross-linking 14% BDDE with 1g of the HA sample (powder, 1 600, 1 100 and 500kDa) | Crosslinking with BDDE resulted in the production of 90-93% water-insoluble HA, with a BDDE/HA content ranging from 4.9-5.3mol%. Cohesivity increased with both increasing molecular weight (MW) and gel concentration in water. Water retention levels were 30ml/g for XHA500 and 75mg/ml for XHA 1600, and gel stiffness increased with higher MW. | “Nutrafast” and Contratto di Sviluppo (Invitalia) | ^11^ |
| Lee, D.Y., 2015 | HA dermal fillers with different HA molecular weight | *In vitro* | Assessing differences in physicochemical properties between HA dermal fillers with different molecular weight of HA. | Hyaluronic acid hydrogels (HA molecular weight - 697, 1058, 1368 kDa) were crosslinked with BDDE (0.1-1 %) to investigate the effect of HA molecular weight on swelling property and elastic modulus of the hydrogels. | The expansion capacity of HA hydrogels rose with increasing the amount of pure HA and was inversely proportional to the crosslinking degree due to an increased number of coiled HA chain interactions. The swelling ratio decreased with increasing the BDDE concentration. | Public (Republic of Korea) | ^12^ |
| Lee, HY., 2015 | Hydrogels crosslinked with polyethylene diglycidyl ether (PEGDE) or BDDE | *In vitro* | Assessing whether PEGDE possesses superior mechanical and chemical properties compared to BDDE by comparing their respective characteristics. | PEGDE or BDDE cross-linked HA hydrogels were compared. A swelling test was performed with fully swollen samples in PBS at 37°C. A rheology measurement was carried out using a parallel plate rheometer. | The rheological behavior of cross-linked HA  shows that PEG-cross-linked HA hydrogels have improved elasticity as compared to BDDE-cross-linked HA at the same molar concentration | No funding to report | ^13^ |
| Li, X., 2021 | HA hydrogel incorporate with different peptides | *In vitro* | The development of a novel HA based hydrogel to be used as a tissue filler and anti-cancer drug delivery vehicle | An anticancer bioactive peptide (ACBP), plant peptide (PB), fishbone collagen (FBC), and bovine bone collagen (BBC) were incorporated into HA hydrogel cross-linked with BDDE as peptide sources and the characteristics of acquired hydrogels were analysed. | All hydrogels reached swelling equilibrium within 4-5 h. The order of swelling ratio (from highest to lowest) in PBS was ACPB Gel > FBC Gel > BBC Gel > PB Gel. The different water absorption capacities were mainly attributed to the hydrophilic groups in the hydrogels. | Public (China) | ^14^ |
| Maiz-Fernández, S., 2019 | HA hydrogels cross-linked with BDDE, divynyl sulfone (DVS) or poly(ethylene glycol) bis (amine)(PEGBNH2) | *In vitro* | Assessing the impact of different crosslinkers on the pH-responsive behavior, particle size, and swelling capacity of HA nanogels and evaluating the effect of varying BDDE content. | HA was crosslinked with BDDE at the following ratios: 1:1, 0.2:1, and 10:1. | A lower BDDE-to-HA ratio (0.2 to 1) promotes swelling in response to both pH changes and transitioning from a dried to a hydrated state. Among all the crosslinkers, BDDE exhibits the highest pH responsiveness. | Public (Basque Country) | ^15^ |
| Schanté, C.E., 2012 | HA -BDDE hydrogels with various amino acids attached to the HA carboxylic group | *In vitro* | Assessing the physical characteristics and improvement of hyaluronic acid enzymatic stability by the grafting of amino acids. | The HA-amino acid products were cross-linked with BDDE. The enzymatic degradation profile in presence of a hyaluronidase and physical characteristics of crosslinked HA were compared to the native HA hydrogels. | All HA-amino acid products have higher swelling ratios than the native HA hydrogel when subjected to the same reaction conditions, suggesting a lower crosslinking density. | No funding to report | ^16^ |
| Shimojo, A.A.M., 2015 | HA microparticles (mHA-BDDE) and sponges (sHA-BDDE) in platelet-rich plasma (prp) | *In vitro* | Evaluating the properties of mHA-BDDE and sHA-BDDE structured HA in PRP | Assessing the rheology, swelling ratio and porosity of acquired HA forms in PRP in comparison to free HA | The results indicate that the microparticle and sponge structures have improved physicochemical and mechanical properties compared to free HA. Structuring HA provides both injectable and solid formulations with superior properties compared to free HA. | Public (Brazil) | ^17^ |
| Venzhik, A.N., 2022 | Gels modified with 0.56mmol of each crosslinking agent (BDDE, PEGDE, PPGDE) | In vitro | Assessing the properties of carboxymethyl cellulose gels modified with BDDE, PEGDE, and PPGDE, and evaluating whether PPDGE serves as a cost-effective alternative to the other crosslinkers. | An NMR analysis of the degree of modification and viscosity was performed | The shear strength of the gel linked with BDDE decreased to 0 when heated to 37°C. The sample made with BDDE has the highest degree of crosslinking, compared to PEDGE and PPGDE. The crosslinked samples have a viscosity higher by 2-3 times in comparison with unmodified CMC. PEDGE is a good alternative crosslinker to BDDE. | No funding to report | ^18^ |
| Yang, B., 2015 | HA crosslinked with BDDE and commercial dermal fillers | *In vitro* | Determining the degree of modification of hyaluronic acid nanogel with BDDE through mass spectroscopy readings. | Mass spectroscopy readings of 1g HA crosslinked with 30,50 and 70ul BDDE + 5 commercial dermal fillers (total n=8) were performed. The total (t-MOD), pendent (p-MOD) and crosslinked (c-MOD) modification were determined. | The t-MOD increased with the amount of BDDE used during preparation, from 2.4% to 4.9%, while the t-MOD in commercial gels was determined to be 0.8% to 5%. The c-MOD to p-MOD ratio in the experimental gels was 75%, which was significantly higher than in commercial gels (13% and 18%).  The study mentions no impact of this measuring method on the properties of the gel. | No funding to report | ^19^ |
| Yang, R., 2016 | HA hydrogels of different BDDE concentrations | *In vitro* | Studying the influence of varying BDDE crosslinker concentrations on the behaviour of HA hydrogels. | The rheological properties of HA hydrogels with the final concentrations of BDDE 0.4 vol%, 0.6 vol%, 0.8 vol% and 1 vol% were investigated. | With the increase in BDDE concentration, the swelling ratio of the resulting HA hydrogels decreased and the rigidity increased correspondingly. At BDDE concentrations higher than 0.8 vol%, this trend was compromised by the BDDE crosslinking efficiency. By varying the BDDE concentration, it becomes possible to create HA hydrogels with viscoelastic properties closely resembling specific tissues. | Public (China) | ^20^ |
| Tang, S., 2021 | Bacterial cellulose and hyaluronic acid gel crosslinked with BDDE | *In vitro* | Determining the rheological properties of bacterial cellulose (BC) crosslinked with hyaluronic acid and BDDE | Bacterial cellulose was crosslinked with 0.1-1% BDDE and a 0.5-2% solution of HA for 24h. Spectroscopy, X-Ray diffraction, mechanical parameter measuring and a thermogravimetric analysis were performed. | The BDDE cross-linked BC/HA composite has better water retention capacity, lower deformation and water solubility than uncross-linked BC/HA | Public (China) | ^21^ |
| Fidalgo, J., 2018 | Hyaluronic acid hydrogels | *In vitro* | Investigating whether alkaline conditions and high temperatures promote the formation of a new byproduct during the BDDE-HA crosslinking reaction. | HA was crosslinked with BDDE at a concentration of 9.9mg/mL and a pH of 12. The resulting gels were filtered and sterilized at a temperature of 120°C. Liquid chromatography and mass spectroscopy (LC-MS) were performed to determine the presence of by-products. | Alkaline conditions and the high temperatures used for sterilizing the HA-BDDE hydrogel promote the formation of a new by-product, resembling "propene glycol”, with an unknown toxicity. This by-product arises under alkaline conditions after autoclaving. | No funding to report | ^22^ |
| Zerbinati, N., 2021 | Cross-linked HA hydrogels   (BDDE) and non-cross-linked HA | In vitro | Exploring the intrinsic matrix organization and rheological properties of various HA-based dermal fillers,  both cross-linked with BDDE and non-cross-linked. | Optical microscopic examination and rheological behavior investigation was performed on various dermal fillers, comparing those crosslinked with BDDE to those without any crosslinker. | The matrix structure, resembling a 'spider web,' is a characteristic feature of cross-linked HA hydrogels. | Matex Lab S.p.A. (author is Scientific Director of Matex Lab) | ^23^ |
| Zhang, J., 2018 | Mice (n=N/A) | In vivo | The development of microneedle patches containing HA crosslinked with BDDE as a means of tissue augmentation | Gels containing HA and BDDE-crosslinked HA (2%) were combined at ratios of 5:1 and 1:1 and compared to a control of uncrosslinked HA in terms of expansion in skin. | The gel which is most crosslinked (1:1) expands least when implanted but only stops expanding at 6 days, compared to 4 days for the 5:1 gel and 2 days for the uncrosslinked gel. | Public (China) | ^24^ |

##

## **Supplementary table 2. HA stability & degradation.**

| **Author & year** | **Population / sample** | **Design** | **Study aim** | **Methodology** | **Key findings** | **Funding** | **Reference** |
| --- | --- | --- | --- | --- | --- | --- | --- |
| Al-Sibani, M., 2016 | Hydrogel 1 and Hydrogel 2 | In vitro | The influence of mixing approach on the properties of BDDE-crosslinked hydrogels | Two cross-linked BDDE-HA hydrogels were prepared by two different mixing approaches; the large-batch mixing approach in which the hydrogel quantities were all mixed as a single lump in one container (hydrogel 1), and the small-batches mixing approach in which the hydrogel quantities were divided into smaller batches, mixed separately at various HA/BDDE ratios then combined in one reaction mixture (hydrogel 2). The degradation rates of cross-linked hydrogels were measured according to a colorimetric method. | Hydrogel 2 exhibited a larger stability against enzymatic digestion and a lower water up-take ability based on the degradation and swelling ratio measurements. | Vivatis Pharma (Hamburg, Germany) supplied the hyaluronic acid. | ^25^ |
| Al-Sibani, M., 2017 | HA hydrogels with different initial HA concentration | *In vitro* | Assessing the impact of different initial concentrations of HA (ranging from 7.0% to 14.0%) on the cross-linking efficiency with 2.0 % BDDE solution and the stability of the hydrogel. | Bovine testicular hyaluronidase (BTH) enzyme with an activity of 300 units /ml was added to each HA hydrogel sample and kept for 2, 3 and 4 days respectively. | 10.0 % HA- based hydrogel proved to be the most stable hydrogel compared to those prepared at either a lower or higher HA concentration. | Vivatis Pharma (Hamburg, Germany) supplied the hyaluronic acid | ^1^ |
| Cui, Y.J., 2012 | HA cross-linked hydrogels (CHA) | *In vitro* | Differentiating the physicochemical properties of various crosslinked hydrogels. | Comparing HA hydrogels cross-linked with BDDE, polyethylene glycol (PEG) poly diglycidyl ether (PDE) or adipic acid dihydrazide (ADH) | While BDDE-crosslinked gels had better enzyme-resistant degradation than ones crosslinked with PEG and PDE, their heat-stability was worse. They remained steady between 20˚C~40˚C, and their stability dropped at 60˚C~100˚C | Public (China) | ^5^ |
| Chang, L. 2019 | HA hydrogels cross-linked with BDDE | *In vitro* | Explore the effects of different BDDE concentrations on HA hydrogel properties. | HA was crosslinked with BDDE at concentrations 5, 10, 15, 20, 25, 30, and 35μL/mL. Anti-enzymatic ability was analysed using hyaluronidase. | Surplus ratio (%) of HA increased with raising BDDE concentrations in a non-linear fashion and decreased over time. | Public (China) | ^3^ |
| Li, X., 2021 | HA hydrogel incorporated with different peptides | *In vitro* | The development of a novel HA based hydrogel for use as a tissue filler and anti-cancer drug delivery vehicle | An anticancer bioactive peptide (ACBP), plant peptide (PB), fishbone collagen (FBC), and bovine bone collagen (BBC) were incorporated to HA hydrogel cross-linked by BDDE as peptide sources and the characteristics of acquired hydrogels were assessed. | Hydrogels crosslinked with BDDE and with added ACBP, PB, FBC, and BBC have high resistance to degradation by collagenase I. | Public (China) | ^14^ |
| Schanté, C.E., 2012 | HA -BDDE hydrogels with various amino acids attached to the HA carboxylic group | *In vitro* | Assessing the physical characteristics and improvement of hyaluronic acid enzymatic stability by the grafting of amino acids. | The HA-amino acid products were cross-linked with BDDE. The enzymatic degradation profile in presence of a hyaluronidase and physical characteristics of crosslinked HA were compared to the native HA hydrogels. | All amino acid-modified HA polymers were more resistant to degradation compared to the native HA albeit with variation according to the amino acids. | No funding to report | ^16^ |
| Zhang, J.N., 2018 | Porcine cadaver skin | *In vitro* | Considering the use of BBDE cross-linked HA (cHA) based microneedles patches (HA-cHA-MNs)  for anti-wrinkle treatment. | A skin test was performed to indicate the insertion ability, in order to compare the mechanical property of different ratios of MNs prepared in this study | The degradation rate decreases with increasing the cHA content in matrix materials of MNs | Public (China) | ^24^ |
| Flégeau K; 2023 | TEOSYAL RHA® fillers (n=4) | In vitro | Determining the degradation kinetics of BDDE-crosslinked fillers | Degradation kinetics in contact with HAase was monitored in real-time by rheological time sweeps. | Repeated administration of small HAase doses (optimal injections every 20 to 30 min) improved HA degradation kinetics over large single doses. | No funding to report (although all authors are employees of Teoxane SA) | ^26^ |
| Baek, J., 2018 | HA hydrogels crosslinked at different temperatures with BDDE    And  Mice (n=10) | *In vitro*  *and*  *In vivo* | Determing the impact of crosslinking HA with BDDE at different temperatures on hydrogel behaviour in vitro and in mice. | The cross-linking temperature ranged from 3°C to 37°C. Properties of obtained HA hydrogels were evaluated in terms of enzymatic degaradation rates of the HA hydrogels in the hyaluronidase solution over 5.5 days. Post injection, the microscopic appearance of surrounding tissues and degradation time were measured at 2, 6 and 10 weeks and compared to a commercial filler. | Cross-linking at a lower temperature greatly improves the enzymatic resistance of HA hydrogels.  After 10 weeks post injection, the HA-BDDE gel (crosslinked at 10° C for 2 weeks) maintains most of its initial volume, whereas the commercial filler is degraded to 40% of its original volume. The HA10 gel had a lower swelling ratio, meaning less hyaluronidase could infiltrate the implant | Public (Korea) | ^2^ |
| Chen, M.A., 2023 | Rabbits (n=N/A) | *In vivo* | Determining the influence of various crosslinking conditions, including BDDE concentration, on the development of polyglutamic acid hydrogels | HA gels were prepared with varying PGA (12-30%) and BDDE (0-30%) concentrations. The gels were implanted into rabbit ears and the degradation rate, swelling and histopathological appearance were examined. | The PGA hydrogel crosslinked with 5% BDDE remained in situ for almost a week longer on average than its un-crosslinked counterpart, but the degradation time decreased when the BDDE concentration was increased to an even higher value. An increase in the concentration of BDDE decreased the necessary time required for PGA gelation. No adverse effects were observed and collagenization was promoted. | Public (China) | ^27^ |
| Hinsenkamp, A.,  2022 | Mice (n=40) | *In vivo* | Developing soft tissue implants using HA crosslinked with BDDE or DVS, supplemented with fibrin | HA gels crosslinked with 2% and 5% BDDE or DVS, with and without fibrin supplementation were injected subcutaneously into mice and the morphology of the tissue was observed at 6 and 12 weeks post implantation. | Gels crosslinked with higher concentrations of BDDE are more durable, as evidenced by a statistically significant decrease in weight of the 2% BDDE scaffold group and no significant decrease in the 5% BDDE group after 12 weeks. The gel crosslinked with DVS is more resistant to degradation than BDDE, making BDDE optimal for soft tissue fillers. | Public (Hungary) | ^28^ |

## **Supplementary table 3. BDDE toxicity**

| **Author & year** | **Population / sample** | **Design** | **Study aim** | **Methodology** | **Key findings** | **Funding** | **Reference** |
| --- | --- | --- | --- | --- | --- | --- | --- |
| Baek, J., 2018 | L929 fibroblasts | *In vitro* | Determing the impact of crosslinking HA with BDDE at different temperatures on hydrogel cytotoxicity. | The cell activity was determined using a methoxyphenyl tetrazolium salt (MTS) assay. After the fibroblast cells were cultured on hydrogels for 5 days, a 10% solution of the assay reagent in complete medium was added to the specimens and incubated at 37 °C for 2 h. Cell attachment and proliferation were evaluated | No cytotoxicity was found. The notably higher in vitro biocompatibility of hydrogels cross-linked in lower temperature was attributed to its higher degree of cross-linking and polymer concentration. | Public (Korea) | ^2^ |
| Choi S.C., 2015 | Fibroblasts | *In vitro* | *Evaluating the in vitro* biocompatibility and cytotoxicity of gels by measuring the degree of inflammatory reaction in fibroblasts | HA gels were prepared with 0.5, 1.0, 5.0 to 10.0 ppm BDDE. The viability of the cells exposed to the the BDDE linked HA hydrogels compared to those of Teflon, Latex and pure HA solutions by thiazoly blue tetrazolium bromide (MTT), neutral reds, and bromodeoxyuridine (BrdU) assays | Addition of higher amounts (10ppm) of BDDE induced higher cell toxicity.. | Public (Republic of Korea) | ^4^ |
| Dias, J.R., 2017 | Human dermal neonatal fibroblasts (hDNF). | *In vitro* | Assessing the toxicity of BDDE crosslinked gelatin towards fibroblasts. | Gelatin was in crosslinked in situ with BDDE at different concentrations (2, 4 and 6 %) and incubation time-points (24, 48 and 72h) at 37ºC. | The crosslinked gelatin presents no toxicity and fibroblasts were able to attach and proliferate, producing new extracellular matrix within the electrospun meshes. | Pubilc (Portugal) | ^6^ |
| Guarise , C., 2019 | BALB/3T3 clone A31 mouse fibroblasts | In vitro | Assessing the impact of the new method of obtaining HA-BDDE on the concentration of BDPE (10,15,30%) , a contaminant formed during crosslinking. | All HBC-hydrogels tested were depolymerized by thermal treatment for 14 h at 100°C, and all the samples tested sterilized by filtration at 0.2 µm.  High performance liquid chromatography (HPLC)-and mass spectrometry (MS) were used to quantify the BDPE content. | Results showed a cytotoxic effect due to the presence of free residual BDPE; the effect was absent when BDPE was chemically linked to HA | No funding to report | ^29^ |
| Jeong, CH., 2021 | Immortalized normal keratinocyte cell line (HaCaT) and human dermal fibroblast cell line (HDF) | *In vitro* | Determining the toxicity of BDDE and PEGDE and assessing the relative safety of these crosslinkers. | HaCaT cells and HDF cells were treated with  BDDE or PEGDE of concentrations 0, 5, 25, 100, 500 and 1000 ppm for 3–24 h. The evaluated parameters were cell viability, cytotoxicity, membrane integrity, reactive oxygen species production (ROS), mitochondrial membrane potential (MMP), and inflammatory responses. | BDDE, at concentrations ranging from 100-1000ppm, significantly reduced cell viability (40% viability) and induced a more substantial LDH release compared to PEGDE. This effect was more pronounced in HaCaT cells and less in HDF cells. BDDE caused cell membrane disruption at 100ppm and elevated ROS production at 10-50ppm in both cell lines. Furthermore, BDDE induced mitochondrial membrane disruption at 10ppm in FDF and 50ppm in HaCaT, which was more pronounced than the effects of PEGDE | Public (Republic of Korea) | ^30^ |
| Kim, DJ., 2021 | Primary culture of human corneal epithelial cells | *In vitro* | Developing a HA membrane (HAM) crosslinked with 0.01% BDDE for treating ocular surface diseases and injuries | Cell viability was assessed by incubation with different concentrations of BDDE for 6 and 48 h and with HAM for 12, 24 and 48h and. Cell viability was measured using CCK-8 analysis. | When applied in vitro, a concentration of 0.1% BDDE had a cytotoxic effect on cells (after 48h, death of 20% of cells was observed). Concentrations of 1 and 10% BDEE killed over 70% of cells. When the crosslinked HAM was applied to cells, cell viability increased by 50% after 48h. | Public (Korea) | ^9^ |
| La Gatta, A., 2020 | Human dermal fibroblast cells | *In vitro* | Modifying the molecular weight of hyaluronic acid and assessing its impact on the properties of HA-BDDE crosslinked hydrogels. | 1g of the HA sample (powder, 1 600, 1 100 and 500kDa) was crosslinked with a 14% BDDE solution. Cell viability was assessed on a human dermal fibroblast cell line after 24h and 48h. | No cytotoxicity was observed after 48h incubation and the expression of marker proteins (collagen I, actin, aqp3) was similar to control. | “Nutrafast” and Contratto di Sviluppo (Invitalia) | ^11^ |
| Lan, SM., 2015 | Schwann cells, rat smooth muscle cells, vascular endothelial cells and NIH/3T3 fibroblast cells | *In vitro* | Studying the impact of perineural application of a HA gel crosslinked with BDDE in terms of biocompatibility and potential adverse effects | Cells were tested for viability against different concentrations  of HA (0.1 and 0.02%), crosslinked HA (0.1 and 0.02%), and dexamethasone (20 mM). The control group had an equal volume of PBS | No adverse effect of native or BDDE-crosslinked HA on the cell viability of smooth muscle cells, vascular endothelial cells, Schwann cells, and fibroblasts up to 72h was observed. | Public (Taiwan) | ^31^ |
| Li, X., 2021 | L929 (mouse fibroblast) and MKN45 (human gastric cancer) cell lines | *In vitro* | The development of a novel HA based hydrogel for use as a tissue filler and anti-cancer drug delivery vehicle | Preliminary cell adhesion tests were carried out to evaluate the feasibility of HA – BDDE cross-linked hydrogels incorporated with anticancer bioactive peptide (ACBP), plant peptide (PB), fishbone collagen (FBC), and bovine bone collagen (BBC) as biomimetic scaffolds for cell survival. | The results showed that the percentage of L929 cell adhesion on all peptide hydrogel samples increased with increasing culture time. In contrast, MKN45 cell adhesion on the hydrogels showed the opposite trend. | Public (China) | ^14^ |
| Shimojo, A.A.M., 2015 | Human adipose tissue-derived mesenchymal stem cells | *In vitro* | Evaluating the effect of microparticles (mHA-BDDE) and sponges (sHA-BDDE) structured HA crosslinked with BDDE in platelet-rich plasma (PRP) on growth factor release and cell proliferation. | Assessing cell compatibility, release of growth factors, cell proliferation and ALP activity. | When combined with PRP, these formulations show promise for bone tissue regeneration. HA-BDDE structures exhibited good compatibility with the cells, enhancing cell proliferation and inducing a remarkable activity of the osteogenic marker alkaline phosphatase | Public (Brazil) | ^17^ |
| Venzhik, A.N., 2022 | MiaPaCa-2 cells | In vitro | Assessing the properties of carboxymethyl cellulose (CMC) gels modified with BDDE, PEGDE, and PPGDE, and evaluating whether PPDGE serves as a cost-effective alternative to the other crosslinkers. | The gels were modified with 0.56mmol of each crosslinking agent (BDDE, PEGDE, PPGDE). A cytotoxicity study was performed using the MTT test (optical density). | The BDDE gel had the lowest cytotoxicity index relative to the control (0.2, compared to 0.6 for other samples.) | No funding to report | ^18^ |
| Xu, C., 2018 | Human dermal fibroblast cells | *In vitro* | Evaluating the effectiveness of 3D printed nanocellulose scaffolds produced by double crosslinking with Ca2+ and BDDE. | Human dermal fibroblast cells were assessed on the common cellular functions (cell attachment, viability, and proliferation on scaffolds) by cell culturing on 2D-plates and various cellulose nanofibril hydrogel 3D-matrices with thicknesses of 2 or 3 mm with a BDDE/ cellulose ratio of 0.01 for low crosslinking and 0.18 for high crosslinking degree. | Fibroblast proliferation was increased at higher BDDE crosslinking level and at higher scaffold thickness. Viability was the same as in the control. | Public (Finland, Australia, China) | ^32^ |
| Yang, R., 2016 | Human fibroblast cells | *In vitro* | Studying the influence of varying BDDE crosslinker concentrations on the properties of HA hydrogels. | The amounts of BDDE residue within HA hydrogels and their cytotoxicity on human fibroblast cells incubated for 2, 4 or 7 days were investigated. The respective viability of cells was then determined by MTT assay. BDDE concentration was 0.4 vol%, 0.6 vol%, 0.8 vol% and 1 vol%. | The relatively easy removal of BDDE residues ensured the suitability of the hydrogels as an injectable scaffold. No cytotoxicity was observed. | Public (China) | ^33^ |
| Del Olmo, J. 2023 | Hyaluronic acid crosslinked with BDDE or DVS  Human dental pulp stem cells  Mice (n=6) | *In vitro and in vivo* | Creating antibacterial coatings based on crosslinked HA on implanted biomaterials made from titanium alloys and determining their antimicrobial potential | Titanium implants were coated with 14%BDDE-HA and 7%DVS-HA and characterized rheologically (swelling ratio, microscopic appearance), as well as in terms of cell toxicity and in vivo toxicity in mice | The implants had a high antimicrobial potential and no cytotoxic effect on cells or in living organisms. The coating crosslinked with BDDE had a lower swelling ability than the one with DVS and a lower ability to release drugs when implanted. | Public (Basque country) | ^34^ |
| Del Olmo, J., 2022 | Hyalurnoc acid crosslinked with different MoDs  Mice (n=10) . | *In vitro and vivo* | Determining the potential of HA gels crosslinked with BDDE for drug delivery and their biocompatibility in vivo | HA was crosslinked with BDDE at concentrations of 0.5, 1, 2M of BDDE. The gels were injected into mice and toxicitiy was determined in terms of clinical symptoms, weight change and general state at 4, 24, 48 and 72h. | The gels are effective vehicles for drug delivery and do not induce any adverse effects in mice, both in terms of general symptoms and at the site of injection | Public (Basque country) | ^35^ |
| National Technical Reports Library - NTIS. Cutaneous Carcinogenicity Study with mice on the diglycidyl ether of 1,4-butane diol with attachments and cover letter dated; 1987 | N=500 (mice) | *In vivo* | Determining the impact of cutaenous BDDE application on the health and survival of mice | 0.05% and 0.2% BDDE in acetone were applied cutaneously to mice for 103 weeks and tissue biopsies were performed and analysed throughout. The types of occuring tumours were recorded. | Topical exposure to BDDE (0.05% and 0.2%) did not affect the survival of mice adversely (30% and 26% of mice surviving in tested groups vs 24% in control) and was not irritant to skin. The cause of their death did not differ from acetone controls. Systemic pathology was not higher in BDDE compared to acetone control. There was no significant increase in number of tumors at the treated sites compared with control, however, a slightly higher number of total tumors of different histogenic origins in BDDE treated group – 5 mice with 6 tumours out of 199 tested compared to 1 mouse with the acetone control. A higher incident of irritant lesions in 0.2% BDDE males (32%) compared to control (14%). | Public  (USA) | ^36^ |
| Kim, M.H., 2021 | Mice (n=N/A) | *In vivo* | Determining the biocompatibility of HA hydrogel crosslinked with BDDE and supplemented with hyaluronidase, and its potential for drug delivery | A HA gel was crosslinked with BDDE in a 1:1.1 ratio and a similar gel containing a hyaluronidase inhibitor was produced. The gels were implanted into mice subcutaneously and the in vivo toxicity was measured after 7 days through blood testing and histological analysis. | BDDE crosslinked hydrogels caused a doubling in the blood urea nitrogen levels (mg/dl) after implantation in mice after 7 days when compared to an uncrosslinked control. No significant histological differences were found between the control, HA and cHA groups (heart, kidney, liver, lung, spleen). Hyaluronidase inhibitors can be an alternative to increasing BDDE concentrations while avoiding toxicity. | Public (Korea) | ^37^ |
| Foureman, P., 1994 | Drosophila melanogaster (n=1841) | *In vivo* | Determining whether BDDE is a mutagenic chemical when applied orally to flies and the types of mutations which occur post application | 1841 flies were fed with 28400 ppm BDDE disolved in water. Their karyotypes were then analysed to observe the types of mutations which occurred. | BDDE is mutagenic with a lethal mutation rate of 1.09%, but it did not cause mortality or sterility in the male flies. A total of 20 lethal mutations occured in the X-chromosomes, A significant number of chromosomal translocations could be recovered in the tested flies. The recovered translocations were T(2:3).  In comparison, 0 mutations occured in the 2462 tests performed on flies which were fed with the solvent control. | Public (USA) | ^38^ |

##

## **Supplementary table 4. BDDE immunogenicity**

| **Author & year** | **Population / sample** | **Design** | **Study aim** | **Methodology** | **Key findings** | **Funding** | **Reference** |
| --- | --- | --- | --- | --- | --- | --- | --- |
| Dewangan, R., 2012 | Rabbits (n=18) | *In vivo* | Studying the effect of various crosslinkers, including BDDE, on the histocompatibility of an acellular matrix impanted subcutaneously | Acellular bladder matrix was crosslinked with 1% BDDE  and compared to grafts produced using other crosslinkers. For each group, grafts with 12/24/48/72h time of incubation with crosslinker implanted in the rabbit, then retrieved after 15, 30, 60 and 90 days. The histopathological score, humoral immune response, and lymphocyte proliferation were analysed. | BDDE crosslinking decreases immunogenicity. The longest crosslinking (72h) time produced the lowest stimulation index of lymphocytes, and 24h time produced highest, but was lower than the control.  The serum response to the BDDE-linked graft grew at day 15, but decreased in all samples at day 30, 60 and returned to baseline level at day 90. | Public (India) | ^39^ |
| Kumar, V., 2015 | Rabbits (n=18) | *In vivo* | Determining the effect of dressing wounds with a swim bladder matrix on tissue healing and a comparison of matrix crosslinked with BDDE to an uncrosslinked control | A swim bladder matrix was crosslinked with BDDE (1%) and compared to a non-crosslinked matrix after implantation for 7, 14, 21 and 28 days in terms of wound healing, immunological response and histological appearance. | The BDDE-linked matrix induces a low inflammatory response. Crosslinking acellular matrix with BDDE decreases serum IgG response at day 20 and 40, back to control level at day 60. The lymphocyte proliferation in the BDDE group was close to control levels | Public (India) | ^40^ |
| Lan, S.M., 2015 | Rats (n=18) | *In vivo* | Studying the impact of perineural application of a HA gel crosslinked with BDDE in terms of biocompatibility and potential adverse effects | HA gels crosslinked with 0.1% and 0.5% BDDE were applied to the excised sciatic nerve. Nerve propagation was measured 4h after treatment and the neurobehaviour of rats and morpohlogy of tissue were analyzed after 2 weeks. | Crosslinking gels with BDDE is not cytotoxic, as demonstrated by no acute or chronic inflammation and no cell necrosis with perineural application of both 0.1% and 0.5% BDDE HA gels at 2 weeks. The gel does not affect neurobehaviour or the propagation of nerve stimuli | Public (Taiwan) | ^31^ |
| Lin, C.L, 2019 | Pigs (n=6) | *In vivo* | Determining whether applying HA crosslinked with BDDE to pigs post-laminectomy has an inhbitory effect on spinal epidural fibrosis formation | A hemi-laminotomy or laminectomy was performed on pigs. Crosslinked HA gel (unknown concentration) was applied, and tissues were analysed after 4 months using MRI, immunohistochemistry and histological examination and compared to an untreated control. | A higher MMP-3 and 9 expression can be seen in the test group, as well as a lower expression of TGF-b and vimentin, increased expression of CD44 and IL-6, and no difference in fibronectin levels. | Public (Taiwan) | ^41^ |
| Geier, J., 2004 | People (n=92) | Observational multicenter study | Assessing the allergenicity of ER system components. | Two patch test series were compiled: the first consisted of ERS components commercially available as patch test substances, and the second contained frequently used components of cold-cured ERS with a presumed or reported allergenic potential. | BDDE together with HDDGE is the most important sensitizer among the reactive diluents used in the production of epoxy resin systems. | Public (Germany) | ^42^ |
| Geier, J., 2016 | People (n=105 656) | Observational retrospective analysis | Exploring concomitant reactivity among reactive diluents and hardeners in epoxy resin system production. | A retrospective analysis of data from the Information Network of Departments of Dermatology (IVDK) regarding the epidemiological surveillance of contact allergy was performed | 144 patients reacted positively to both chemicals, representing 92.9% of those reacting to BDDE and 75.4% of those reacting to HDDE. | Public (Germany) | ^43^ |
| Jolanki, R., 1987 | People (n=3) | Case-series study | Assessing the sensitizing strength of BDDGE. | The patients were patch tested using Finn-chamber on the upper back, with an occlusion time of 24h. Permeation studies were also performed to provide information about permeability of gloves to BDDE. | BDDGE may be an even stronger sensitizer in humans than epoxy resin.  The glove material was more permeable to the reactive diluent than to epoxy resin, leading to sensitivity to BDDGE rather than epoxy resin. | No funding to report | ^44^ |
| Aalto-Korte, K., 2015 | People (n=67) | Retrospective observational study | Investigating the incidence and clinical significance of allergic reactions to epoxy diluents and epoxy resins. | Files from 1991 to 2014 were examined, and the clinical records of patients exhibiting allergic reactions were scrutinized for occupation, concurrent allergic responses, and exposure. | In 3 cases, 1,4-butanediol diglycidyl ether was the sole cause of occupational allergic contact dermatitis. | Public (Finland) | ^45^ |
| Decates, T., 2021 | People (n=12) | Prospective clinical study | Investigating the role of HA-fillers in inducing delayed-type hypersensitivity. | Twelve patients underwent comprehensive allergic screening, including patch tests and specific intradermal testing on the medial upper arm. Various commercially available hyaluronic acid (HA) fillers were injected in 0.1 cc boluses, and a positive allergic reaction was characterized by erythema, firmness, or swelling. | The findings suggest that late inflammatory reactions (LIRs) to hyaluronic acid (HA) fillers are not associated with either type I or type IV hypersensitivity, but with the degradation of cross-linked HA filler. This may expose trace amounts of BDDE, bacterial proteins and low molecular weight HA to exert a direct pro-inflammatory reaction. | No funding to report | ^46^ |

## **Supplementary table 5. BDDE-tissue interactions – including enhancing proliferation, inflammation modulation etc.**

| **Author & year** | **Population / sample** | **Design** | **Study aim** | **Methodology** | **Key findings** | **Funding** | **Reference** |
| --- | --- | --- | --- | --- | --- | --- | --- |
| Bang, S., 2017 | MC3T3 osteoblast cel line | In vitro | Evaluating the suitability of an HA-BDDE gel scaffold for efficient bone regeneration, assessing its impact on osteoblast cells, and examining its capacity for controlled drug release (DMOG). | The cellular behavior of osteoblast was evaluated in the presence and absence of dimethyloxalylglycine (DMOG) and sodium borate (NaB) in the pattern gel (HA-BDDGE) by conducting swelling study, loading and release study, cell cultures, live and dead assay, ALP assay and protein expressions of HIF-1α and Runx2 using western blot assay. | The BDDE-HA crosslinked gel exhibited organized, uniform patterning and biocompatibility with MC3T3 (osteoblast) lines. Cell proliferation and expansion on the scaffold were observed, and cell-to-cell contact was enhanced by the gel's organized nature, as confirmed by staining imaging. The gel also demonstrated controlled release of the DMOG drug. | Public (Republic of Korea) | ^47^ |
| Tang, S., 2021 | [Mouse](https://www-1sciencedirect-1com-10000376r002d.han3.wum.edu.pl/topics/agricultural-and-biological-sciences/mus-musculus) fibroblast (L929) cells | *In vitro* | Determining whether bacterial cellulose (BC) crosslinked with hyaluronic acid and BDDE promotes cell adhesion and proliferation | Bacterial cellulose was crosslinked with 0.1-1% BDDE and a 0.5-2% solution of HA for 24h. The effect of the crosslinked cellulose on cells was measured using fluorescent and scanning electron microscopy. | When crosslinked with BDDE, the composites had improved cell proliferation and adhesion. This may be due to the increased roughness of the material. A positive effect on cell viability was noted. | Public (China) | ^21^ |
| Baek, J., 2018 | Mice (n=10) | In vivo | Determing the impact of crosslinking HA with BDDE at different temperatures on hydrogel behaviour | Hyaluronic acid was crosslinked with 2%v/v% BDDE at 37 °C, 20°C, 10 °C, or 3 °C. Post injection, the microscopic appearance of surrounding tissues and degradation time were measured at 2, 6 and 10 weeks. | A fibrous capsule forms around the gel, decreasing hyaluronidase infiltration.  BDDE crosslinked at lower temperatures induces greater tissue regeneration and thicker dermis than a commercially available control, with 75% of the dermis layer consisting of collagen compared to 55% in the control and elastic fibers forming 14% vs 11% of the area of implantation. | Public (Korea) | ^2^ |
| Guarise, C., 2019 | Rabbits (n=5) | *In vivo* | Developing a new method for high-purity BDDE-crosslinked HA filler synthesis and determining the best predictor of a filler’s clinical performance | HA was crosslinked with 10, 15 and 30mol/% BDDE and was injected into mice subcutaneously after storage at 55°C for 14, 30, and 45 days. The degradation rate and potential adverse reactions were observed. | After having been stored for 30 days at 55°C, the BDDE-crosslinked hyaluronan had a stable degradation rate over a two-month observation period. After 45 days of storage, the degradation rate increased almost twofold. Elasticity is the best predictor of a gel’s degradation time in vivo, as both parameters follow the same trend. No adverse reactions occurred. | No funding to report | ^29^ |
| Hinsenkamp, A., 2022 | Mice (n=40) | *In vivo* | Developing soft tissue implants using HA crosslinked with BDDE or DVS, supplemented with fibrin | HA gels crosslinked with 2% and 5% BDDE or DVS, with and without fibrin supplementation were injected subcutaneously into mice and the morphology of the tissue was observed at 6 and 12 weeks post implantation. | Crosslinking with BDDE enables better cell infiltration and ECM integration than DVS, which is amplified by adding fibrin Vascularization is higher in all BDDE samples compared to DVS and in higher BDDE concentrations. 5% BDDE-HA induced area of RBCs of 2% after 12 weeks, compared to 0.3% in 2% BDDE. | Public (Hungary) | ^48^ |
| Kim, D.J., 2021 | Rabbits (n=30) | *In vivo* | Developing a HA membrane crosslinked with BDDE for treating ocular surface diseases and injuries | HA was crosslinked with BBDE at a concentration of 0.01%. Different wound models were created on rabbit eyes and the test membrane was applied together. Wound healing and mRNA expression for fibrotic gene (vimentin and smooth muscle actin) expression were analyzed and compared to an untreated control. | The HA membrane is an effective medium of aiding tissue regeneration, by inducing complete re-epithelialization after 72h, compared to 82.8% in the control. In the scar and burn models, applying the HAM normalizes the fibrotic gene expression, decreases the neovascularization and increases the transparency of the tissues. | Public (Korea) | ^9^ |
| Kumar, V., 2015 | Rabbits (n=18) | *In vivo* | Determining the effect of dressing wounds with a swim bladder matrix on tissue healing and a comparison of matrix crosslinked with BDDE to an uncrosslinked control | A swim bladder matrix was crosslinked with BDDE (1%) and compared to a non-crosslinked matrix after implantation for 7, 14, 21 and 28 days in terms of wound healing, immunological response and histological appearance. | Crosslinking with BDDE enhances wound healing and decreases scarring. On day 7 and 14 histopathological scores were better for ABSM-BDDE when it comes to collagen arrangement and density, reaching an equal level to the control by day 21. Complete healing was observed after day 21 with no scarring and circa 15% contraction. | Public (India) | ^40^ |
| Lan, S.M., 2021 | Rats (n=18) | *In vivo* | Studying the impact of perineural application of a HA gel crosslinked with BDDE in terms of biocompatibility and potential adverse effects | HA gels crosslinked with 0.1% and 0.5% BDDE were applied to the excised sciatic nerve. Nerve propagation was measured 4h after treatment and the neurobehaviour of rats and morpohlogy of tissue were analyzed after 2 weeks. | 0.5% BDDE-HA induced a higher rate of epineural vascularization than 0.1% BDDE and control groups, with no differences in intraneural levels. . A slight viscosity of BDDE crosslinked HA gels was observed. | Public (Taiwan) | ^31^ |
| Li, X., 2021 | Mice (n=30) | *In vivo* | The development of a HA hydrogel crosslinked with BDDE, supplemented with collagen, for use as a tissue filler and anti-cancer drug delivery vehicle | A HA gel was crosslinked with ca. 6% BDDE and infused with various biological anti-tumor peptides. The gel was implanted into mice with a tumor model and the tumor size and its morphology were analysed after two weeks. | The hydrogels are an effective means of drug delivery and have a tumor-decreasing effect on the mice. | Public (China) | ^14^ |
| Lin, C.L, 2019 | Pigs (n=6) | *In vivo* | Determining whether applying HA crosslinked with BDDE to pigs post-laminectomy has an inhbitory effect on spinal epidural fibrosis formation | A hemi-laminotomy or laminectomy was performed on pigs. Crosslinked HA gel (unknown concentration) was applied, and tissues were analysed after 4 months using MRI, immunohistochemistry and histological examination and compared to an untreated control. | The BDDE-HA gel results in a better organization of ECM, with less collagen and elastic fiber deposition. | Public (Taiwan) | ^41^ |
| Yang, R., 2016 | Mice (n=N/A) | *In vivo* | Studying the behaviour of BDDE crosslinked HA hydrogel in vivo and its potential for cell delivery | HA was crosslinked with 0.4 vol/% BDDE. The gels were used as a scaffold for cartilage and dental tissue and implanted. The samples were analysed at 15 and 36 weeks post implantation histologically and using immunochemistry. | The resulting gels can serve as an effective vehicle for cell delivery. Subcutaneous injection of HA-BDDE scaffold did not induce tissue growth except for fibrous encapsulation | Public (China) | ^33^ |
| Yeom, J., 2010 | Mice (n=24) | *In vivo* | Developing a novel filler containing HMDA and comparing it to fillers containing commonly used crosslinkers, like BDDE and DVS | HMDA-crosslinked HA gels were implanted subcutaneously to nude mice and compared with Restylane, a commercial BDDE-linked filler containing ca. 3-5%/mol BDDE in terms of dermal thickness and collagen density. | The BDDE- crosslinked filler induces significant tissue regeneration, with a 30% thicker dermis layer and a 20% increase in collagen density compared to negative control.The lymphocyte infiltration rate at the site of application is significantly higher than when HMDA is used. | Public (Korea) | ^49^ |
| Hsu, DZ., 2016 | Primary rat tenocytes (from Achilles tendon) | *In vivo* | Investigating the influence of crosslinking HA with BDDE on preventing fibrosis | 0-0.25% crosslinked hyaluronic acid was applied to primary tenocyte cultures and the levels of various protein expression, cell proliferation, migration and autophagy were determined. | HA-crosslinked with BDDE may have the potential for preventing epidural fibrosis, as opposed to uncrosslinked HA. This is done by inhibtiing cell migration and proliferation, decreasing fibrotic gene expression and increasing autophagy. | Public (Taiwan) | ^50^ |
| Decates, T., 2021 | People (n=12) | Prospective clinical study | Investigating the role of HA-fillers in inducing delayed-type hypersensitivity. | Twelve patients underwent comprehensive allergic screening, including patch tests and specific intradermal testing on the medial upper arm. Various commercially available hyaluronic acid (HA) fillers were injected in 0.1 cc boluses, and a positive allergic reaction was characterized by erythema, firmness, or swelling. | The findings indicate that late inflammatory reactions (LIRs) to hyaluronic acid (HA) fillers are not associated with either type I or type IV hypersensitivity, but with the degradation of cross-linked HA filler, this may expose trace substance of BDDE, bacterial proteins and low molecular weight HA to exert a direct pro-inflammatory reaction. | No funding to report | ^46^ |

## **Supplementary table 6. Clinical data on BDDE-crosslinked HA**

| **Author & year** | **Population / sample** | **Design** | **Study aim** | **Methodology** | **Key findings** | **Funding** | **Reference** |
| --- | --- | --- | --- | --- | --- | --- | --- |
| Aalto-Korte, K., 2015 | People (n=67) | Retrospective observational study | Investigating the incidence and clinical significance of allergic reactions to epoxy diluents and epoxy resins. | Files from 1991 to 2014 were examined, and the clinical records of patients exhibiting allergic reactions were scrutinized for occupation, concurrent allergic responses, and exposure. | In 3 cases, 1,4-butanediol diglycidyl ether was the sole cause of occupational allergic contact dermatitis. | Public (Finland) | ^45^ |
| Decates, T., 2021 | People (n=12) | Prospective clinical study | Investigating the role of HA-fillers in inducing delayed-type hypersensitivity. | Twelve patients underwent comprehensive allergic screening, including patch tests and specific intradermal testing on the medial upper arm. Various commercially available hyaluronic acid (HA) fillers were injected in 0.1 cc boluses, and a positive allergic reaction was characterized by erythema, firmness, or swelling. | The findings indicate that late inflammatory reactions (LIRs) to hyaluronic acid (HA) fillers are not associated with either type I or type IV hypersensitivity, but with the degradation of cross-linked HA filler, this may expose trace substance of BDDE, bacterial proteins and low molecular weight HA to exert a direct pro-inflammatory reaction. | No funding to report | ^46^ |
| Geier, J., 2004 | People (n=92) | Observational multicenter study | Assessing the allergenicity of ER system components. | Two patch test series were compiled: the first consisted of ERS components commercially available as patch test substances, and the second contained frequently used components of cold-cured ERS with a presumed or reported allergenic potential. | BDDE together with HDDGE is the most important sensitizer among the reactive diluents used in the production of epoxy resin systems. | Public (Germany) | ^42^ |
| Geier, J., 2016 | People (n=105 656) | Observational retrospective analysis | Exploring concomitant reactivity among reactive diluents and hardeners in epoxy resin system production. | A retrospective analysis of data from the Information Network of Departments of Dermatology (IVDK) regarding the epidemiological surveillance of contact allergy | 144 patients reacted positively to both chemicals, representing 92.9% of those reacting to BDDE and 75.4% of those reacting to HDDE. | Public (Germany) | ^43^ |
| Jolanki, R., 1987 | People (n=3) | Case-series study | Assessing the sensitizing strength of BDDGE. | The patients were patch tested using Finn-chamber method on the upper back, with an occlusion time of 24h. Also, there were performed permeation studies to get information about permeability of gloves to BDDE. | BDDE may be an even stronger sensitizer in humans than epoxy resin.  The glove material was more permeable to the reactive diluent than to epoxy resin, leading to sensitivity to BDDGE rather than epoxy resin. | No funding to report | ^44^ |
| Rzany, B., 2019 | People (n=90) | Prospective, monocentric, double-blinded, randomized, controlled trial | Evaluating dermal fillers with reduced BDDE content as a viable alternative to commercially available fillers. | A double-blinded, randomized, controlled trial was conducted on 90 subjects with moderate to severe bilateral nasolabial folds (NLFs). The prospective, monocentric study, implemented a split-face design and evaluated efficacy parameters over 18 months. Assessment included improvement on the Wrinkle Severity Rating Scale, Global Aesthetic Improvement Scale, and quantitative imaging for NLF correction. | There was no statistcial difference between gels in terms of global aestethic improvements and injectors were satisfied with both, however, resilient hyaluronic acid gels had higher appraisal scores for each category (easiness of injection, of product positioning, results produced). | TEOXANE, SA,  Geneva, Switzerland | ^51^ |
| Ji, GY., 2015 | People  (n=68) | *Prospective, multicenter, double-blinded and randomized trial* | Comparing the influence of sodium hyaluronated crosslinked with BDDE as opposed to with sodium carboxymethylcellulose (CMC) on preventing adhesion formation after lumbar discectomy | Subjects were randomly divided into two groups, receiving either CMC or BDDE crosslinked hyaluronan before wound closure after a discectomy. 3 and 6 weeks after surgery, the results were evaluated in terms of a visual morphology scale, a disability assessment and radiological outcomes measured using MRI | BDDE is more effective in stabilising HA than CMC, resulting in better wound healing and lower pain at 3 and 6 weeks post-surgery. However, after BDDE-HA implantation there was a statistically significant increased freuqency of adverse effects related to the nervous system, such as nerve root irritation and hypoesthesia. | Cha Bio and Diostech | ^52^ |

**References:**

1. Al-Sibani M, Al-Harrasi A, Neubert RHH. Effect of hyaluronic acid initial concentration on cross-linking efficiency of hyaluronic acid - based hydrogels used in biomedical and cosmetic applications. Pharmazie. 2017;72; 81-86.

2. Baek J, Fan Y, Jeong SH, Lee HY, Jung HD, Kim HE, et al. Facile strategy involving low-temperature chemical cross-linking to enhance the physical and biological properties of hyaluronic acid hydrogel. Carbohydr Polym. 2018;202; 545-553.

3. Chang L, Zhang J, Jiang X. Comparative Properties of Hyaluronic Acid Hydrogel Cross-linked with 1, 4-Butanediol Diglycidyl Ether Assayed Using a Marine Hyaluronidase. IOP Conference Series: Materials Science and Engineering. IOP Publishing, 2019. 012007.

4. Choi SC, Yoo MA, Lee SY, Lee HJ, Son DH, Jung J, et al. Modulation of biomechanical properties of hyaluronic acid hydrogels by crosslinking agents. J Biomed Mater Res A. 2015;103; 3072-3080.

5. Cui YJ, Wang WG, Li P, Zhao YL, Gu YN, Wan JL. The Comparison of Physicochemical Properties of Four Cross-Linked Sodium Hyaluronate Gels with Different Cross-Linking Agents. Adv Mat Res. 2012;396-398; 1506-1512.

6. Dias J, Baptista da Silva S, Oliveira C, Sousa A, Oliveira A, Bártolo P, Granja P. In situ crosslinked electrospun gelatin nanofibers for skin regeneration. Eur Polym J. 2017;95; 161-173.

7. Kaya G, Oytun F. Rheological Properties of İnjectable Hyaluronic Acid Hydrogels for Soft Tissue Engineering Applications. Biointerface Res Appl Chem. 2021;11; 8424-8430.

8. Keizers PHJ, Vanhee C, van den Elzen EMW, de Jong WH, Venhuis BJ, Hodemaekers HM, et al. A high crosslinking grade of hyaluronic acid found in a dermal filler causing adverse effects. J Pharm Biomed Anal. 2018;159; 173-178.

9. Kim DJ, Jung MY, Pak HJ, Park JH, Kim M, Chuck RS, Park CY. Development of a novel hyaluronic acid membrane for the treatment of ocular surface diseases. Sci Rep. 2021;11; 2351.

10. Kumar V, Kumar N, Singh H, Gangwar A, Dewangan R, Kumar A, Rb R. Effects of crosslinking treatments on the physical properties of acellular fish swim bladder. Trends in Biomaterials and Artificial Organs. 2013;27; 93-101.

11. La Gatta A, Salzillo R, Catalano C, Pirozzi AVA, D'Agostino A, Bedini E, et al. Hyaluronan-based hydrogels via ether-crosslinking: Is HA molecular weight an effective means to tune gel performance? Int J Biol Macromol. 2020;144; 94-101.

12. Lee D, Cheon C, Son S, Kim Y-Z, Kim J-T, Jang J-W, Kim S-S. Influence of Molecular Weight on Swelling and Elastic Modulus of Hyaluronic Acid Dermal Fillers. Polymer Korea. 2015;39; 976-980.

13. Lee H-y, Song J, Kim HE. Mechanical Improvement of Hyaluronic Acid ( HA ) Hydrogels and Incorporation of Polyethylene Glycol ( PEG ). Society for Biomaterials, 2015.

14. Li X, Cui H, Suyila Q, Yang X, Wu X, Su X. The hydrogels based on peptide/collagen as potential multifunctional materials for soft tissue filling and inhibition of tumor growth. Int J Polym Mater. 2021;71; 1-13.

15. Maiz-Fernandez S, Perez-Alvarez L, Ruiz-Rubio L, Perez Gonzalez R, Saez-Martinez V, Ruiz Perez J, Vilas-Vilela JL. Synthesis and Characterization of Covalently Crosslinked pH-Responsive Hyaluronic Acid Nanogels: Effect of Synthesis Parameters. Polymers (Basel). 2019;11.

16. Schante C, Zuber G, Herlin C, Vandamme T. Improvement of hyaluronic acid enzymatic stability by the grafting of amino-acids. Carbohydr Polym. 2012;87; 2211-2216.

17. Shimojo A, Galdames S, Duarte ADSS, Pina LM, Rodrigues A, Luzo A, Santana MH. The structuring of high molecular weight hyaluronic acid in microparticles or sponges improves its performance when associated with platelet-rich plasma. Trends Biomater Artif Organs. 2015;29; 160-169.

18. Venzhik AN, Nikolaev DA, Romanova IV. Study of Rheological and Structural Properties of Modified Carboxymethyl Cellulose Solutions Using Crosslinking Agents Based on Substituted Oxyranes. Inorg Mater Appl Res. 2022;13; 378-385.

19. Yang B, Guo X, Zang H, Liu J. Determination of modification degree in BDDE-modified hyaluronic acid hydrogel by SEC/MS. Carbohydr Polym. 2015;131; 233-239.

20. Yang R, Tan L, Cen L, Zhang Z. An injectable scaffold based on crosslinked hyaluronic acid gel for tissue regeneration. RSC Advances. 2016;6; 16838-16850.

21. Tang S, Chi K, Xu H, Yong Q, Yang J, Catchmark JM. A covalently cross-linked hyaluronic acid/bacterial cellulose composite hydrogel for potential biological applications. Carbohydr Polym. 2021;252; 117123.

22. Fidalgo J, Deglesne PA, Arroyo R, Sepulveda L, Ranneva E, Deprez P. Detection of a new reaction by-product in BDDE cross-linked autoclaved hyaluronic acid hydrogels by LC-MS analysis. Med Devices (Auckl). 2018;11; 367-376.

23. Zerbinati N, Sommatis S, Maccario C, Capillo MC, Grimaldi G, Alonci G, et al. Comparative Physicochemical Analysis among 1,4-Butanediol Diglycidyl Ether Cross-Linked Hyaluronic Acid Dermal Fillers. Gels. 2021;7.

24. Zhang J, Chen BZ, Ashfaq M, Zhang X, Guo XD. Development of a BDDE-crosslinked hyaluronic acid based microneedles patch as a dermal filler for anti-ageing treatment. J Ind Eng Chem. 2018;65.

25. Al-Sibani M, Al-Harrasi A, Neubert RH. Study of the effect of mixing approach on cross-linking efficiency of hyaluronic acid-based hydrogel cross-linked with 1,4-butanediol diglycidyl ether. Eur J Pharm Sci. 2016;91; 131-137.

26. Flégeau K, Jing J, Brusini R, Gallet M, Moreno C, Walker L, et al. Multidose Hyaluronidase Administration as an Optimal Procedure to Degrade Resilient Hyaluronic Acid Soft Tissue Fillers. Molecules. 2023;28.

27. Chen M, Chen L, Yuan D, Niu L, Hu J, Zhang X, et al. Preparation, function, and safety evaluation of a novel degradable dermal filler, the cross-linked poly-gamma-glutamic acid hydrogel particles. J Biomed Mater Res B Appl Biomater. 2023;111; 1407-1418.

28. Hinsenkamp A, Fulop A, Hricisak L, Pal E, Kun K, Majer A, et al. Application of Injectable, Crosslinked, Fibrin-Containing Hyaluronic Acid Scaffolds for In Vivo Remodeling. J Funct Biomater. 2022;13.

29. Guarise C, Barbera C, Pavan M, Panfilo S, Beninatto R, Galesso D. HA-based dermal filler: downstream process comparison, impurity quantitation by validated HPLC-MS analysis, and in vivo residence time study. J Appl Biomater Funct Mater. 2019;17; 2280800019867075.

30. Jeong CH, Kim DH, Yune JH, Kwon HC, Shin DM, Sohn H, et al. In vitro toxicity assessment of crosslinking agents used in hyaluronic acid dermal filler. Toxicol In Vitro. 2021;70; 105034.

31. Lan SM, Jou IM, Wu PT, Wu CY, Chen SC. Investigation into the safety of perineural application of 1,4-butanediol diglycidyl ether-crosslinked hyaluronan in a rat model. J Biomed Mater Res B Appl Biomater. 2015;103; 718-726.

32. Xu C, Zhang Molino B, Wang X, Cheng F, Xu W, Molino P, et al. 3D printing of nanocellulose hydrogel scaffolds with tunable mechanical strength towards wound healing application. J Mater Chem B. 2018;6; 7066-7075.

33. Yang R, Tan L, Cen L, Zhang Z. An injectable scaffold based on crosslinked hyaluronic acid gel for tissue regeneration. RSC Adv. 2016;6; 16838-16850.

34. Andrade del Olmo J, Alonso JM, Sáez-Martínez V, Benito-Cid S, Pérez-González R, Vilas-Vilela JL, Pérez-Álvarez L. Hyaluronic acid-based hydrogel coatings on Ti6Al4V implantable biomaterial with multifunctional antibacterial activity. Carbohydr Polym. 2023;301; 120366.

35. Andrade Del Olmo J, Perez-Alvarez L, Saez Martinez V, Benito Cid S, Perez Gonzalez R, Vilas-Vilela JL, Alonso JM. Drug Delivery from Hyaluronic Acid-BDDE Injectable Hydrogels for Antibacterial and Anti-Inflammatory Applications. Gels. 2022;8.

36. CIBA-GEIGY Corp. A, NY., Environmental Protection Agency W, DC. Office of Toxic. Cutaneous Carcinogenicity Study with Mice on the Diglycidyl Ether of 1,4-butanediol with Attachments and Cover Letter dated 09/28/1987. 1987. 53.

37. Kim MH, Park JH, Nguyen DT, Kim S, Jeong DI, Cho HJ, Kim DD. Hyaluronidase Inhibitor-Incorporated Cross-Linked Hyaluronic Acid Hydrogels for Subcutaneous Injection. Pharmaceutics. 2021;13.

38. Foureman P, Mason JM, Valencia R, Zimmering S. Chemical mutagenesis testing in Drosophila. IX. Results of 50 coded compounds tested for the National Toxicology Program. Environ Mol Mutagen. 1994;23; 51-63.

39. Dewangan R, Sharma AK, Kumar N, Maiti S, Singh H, Gangwar A, et al. In-vitro biocompatibility determination of bladder acellular matrix graft. Trends Biomater Artif Organs. 2012;25; 161-171.

40. Kumar V, Kumar N, Gangwar AK, Singh H, Singh R. Comparative histologic and immunologic evaluation of 1,4-butanediol diglycidyl ether crosslinked versus noncrosslinked acellular swim bladder matrix for healing of full-thickness skin wounds in rabbits. Journal of Surgical Research. 2015;197; 436-446.

41. Lin CL, Jou IM, Wu CY, Kuo YR, Yang SC, Lee JS, et al. Topically Applied Cross-Linked Hyaluronan Attenuates the Formation of Spinal Epidural Fibrosis in a Swine Model of Laminectomy. Sci Rep. 2019;9; 14613.

42. Geier J, Lessmann H, Hillen U, Jappe U, Dickel H, Koch P, et al. An attempt to improve diagnostics of contact allergy due to epoxy resin systems. First results of the multicentre study EPOX 2002. Contact Dermatitis. 2004;51; 263-272.

43. Geier J, Lessmann H, Hillen U, Skudlik C, Jappe U. Sensitization to reactive diluents and hardeners in epoxy resin systems. IVDK data 2002-2011. Part II: concomitant reactions. Contact Dermatitis. 2016;74; 94-101.

44. Jolanki R, Estlander T, Kanerva L. Contact allergy to an epoxy reactive diluent: 1,4-butanediol diglycidyl ether. Contact Dermatitis. 1987;16; 87-92.

45. Aalto-Korte K, Kuuliala O, Henriks-Eckerman ML, Suuronen K. Contact allergy to reactive diluents and related aliphatic epoxy resins. Contact Dermatitis. 2015;72; 387-397.

46. Decates T, Kadouch J, Velthuis P, Rustemeyer T. Immediate nor Delayed Type Hypersensitivity Plays a Role in Late Inflammatory Reactions After Hyaluronic Acid Filler Injections. Clin Cosmet Investig Dermatol. 2021;14; 581-589.

47. Bang S, Das D, Yu J, Noh I. Evaluation of MC3T3 Cells Proliferation and Drug Release Study from Sodium Hyaluronate-1,4-butanediol Diglycidyl Ether Patterned Gel. Nanomaterials (Basel). 2017;7.

48. Hinsenkamp A, Fülöp Á, Hricisák L, Pál É, Kun K, Majer A, et al. Application of Injectable, Crosslinked, Fibrin-Containing Hyaluronic Acid Scaffolds for In Vivo Remodeling. J Funct Biomater. 2022;13.

49. Yeom J, Bhang SH, Kim BS, Seo MS, Hwang EJ, Cho IH, et al. Effect of cross-linking reagents for hyaluronic acid hydrogel dermal fillers on tissue augmentation and regeneration. Bioconjug Chem. 2010;21; 240-247.

50. Hsu DZ, Jou IM. 1,4-Butanediol diglycidyl ether-cross-linked hyaluronan inhibits fibrosis in rat primary tenocytes by down-regulating autophagy modulation. J Mater Sci Mater Med. 2016; 27(25):84.

51. Rzany B, Converset-Viethel S, Hartmann M, Larrouy JC, Ribe N, Sito G, Noize-Pin C. Efficacy and Safety of 3 New Resilient Hyaluronic Acid Fillers, Crosslinked With Decreased BDDE, for the Treatment of Dynamic Wrinkles: Results of an 18-Month, Randomized Controlled Trial Versus Already Available Comparators. Dermatol Surg. 2019;45; 1304-1314.

52. Ji GY, Oh CH, Moon BG, Yi S, Han IB, Heo DH, et al. Efficacy and Safety of Sodium Hyaluronate with 1,4-Butanediol Diglycidyl Ether Compared to Sodium Carboxymethylcellulose in Preventing Adhesion Formation after Lumbar Discectomy. Korean J Spine. 2015;12(2); 41-47.
